# Optimal Cut-Off Value of the Superior Articular Process Area as a Morphological Parameter to Predict Lumbar Foraminal Stenosis

**DOI:** 10.1155/2017/7914836

**Published:** 2017-01-09

**Authors:** Tae-Ha Lim, Soo Il Choi, Hyung Rae Cho, Keum Nae Kang, Chang Joon Rhyu, Eun Young Chae, Young Su Lim, Yongsoo Lee, Young Uk Kim

**Affiliations:** ^1^Department of Anesthesiology and Pain Medicine, Eulji General Hospital, Eulji University College of Medicine, Seoul, Republic of Korea; ^2^Department of Anesthesiology and Pain Medicine, Catholic Kwandong University of Korea College of Medicine, International St. Mary's Hospital, Incheon, Republic of Korea; ^3^Department of Anesthesiology and Pain Medicine, Myongji Hospital, College of Medicine, Seonam University, Goyang, Republic of Korea; ^4^Department of Anesthesiology and Pain Medicine, National Police Hospital, Seoul, Republic of Korea; ^5^Department of Radiology, Asan Medical Center, University of Ulsan College of Medicine, Seoul, Republic of Korea

## Abstract

*Background*. We devised a new morphological parameter called the superior articular process area (SAPA) to evaluate the connection between lumbar foraminal stenosis (LFS) and the superior articular process.* Objective*. We hypothesized that the SAPA is an important morphologic parameter in the diagnosis of LFS.* Methods*. All patients over 60 years of age were included. Data regarding the SAPA were collected from 137 patients with LFS. A total of 167 control subjects underwent lumbar magnetic resonance imaging (MRI) as part of a routine medical examination. We analyzed the cross-sectional area of the bone margin of the superior articular process at the level of L4-L5 facet joint in the axial plane.* Results*. The average SAPA was 96.3 ± 13.6 mm^2^ in the control group and 128.1 ± 17.2 mm^2^ in the LFS group. The LFS group was found to have significantly higher levels of SAPA (*p* < 0.001) in comparison to the control group. In the LFS group, the optimal cut-off value was 112.1 mm^2^, with 84.4% sensitivity, 83.9% specificity, and AUC of 0.94 (95% CI: 0.91–0.96).* Conclusions*. Higher SAPA values were associated with a higher possibility of LFS. These results are important in the evaluation of patients with LFS.

## 1. Introduction

Lumbar spinal stenosis (LSS) results from degenerative changes in the spinal canal and is the most common spinal disease in elderly individuals [1–3]. It typically presents with buttock or low back pain, sensory and motor disturbances in the lower leg, and neurogenic intermittent claudication [2, 4]. LSS is characterized by narrowing of the spinal canal, which is caused by disc herniation combined with osteophytes, hypertrophy of the ligamentum flavum, and mechanical compression of the spinal nerve roots [5, 6]. Facet joint hypertrophy is considered another major cause of LSS [7]. Anatomically, degenerative LSS can involve the central canal, the foramina, the extraforaminal zone, or a combination of these locations. Lumbar foraminal stenosis (LFS) is defined as when the spinal nerve roots are compressed on the side due to narrowing of the foramen that may be caused by a foraminal herniated disc, a collapsed disc space, or an enlarged joint [6]. The foramen can be narrowed further by characteristic changes in the facet joints such as synovial cysts, osteoarthritis, or hypertrophy of articular processes [8, 9]. All of the changes contribute to LFS symptoms [4, 5]. Previous studies have indicated that morphologic parameters including the dural sac area, spinal canal area, and ligamentum flavum thickness are associated with disc degeneration, aging, and LSS [10–12]. However, few studies have actually examined how facet joint hypertrophy affects LFS. The cross-sectional area of the superior articular process is an important morphologic parameter in the identification of facet joint hypertrophy [7]. Barry and Livesley measured the cross-sectional area of the superior articular process in the transverse plane using computed tomography [7]. However, they did not evaluate the role of SAPA as a morphological parameter of LFS. Therefore, in order to evaluate the connection between LFS and hypertrophy of the superior articular process, we devised a new morphological parameter called the superior articular process area (SAPA). The SAPA has not yet been evaluated for its association with LFS. We hypothesized that the SAPA is an important morphologic parameter in the diagnosis of LFS. Therefore, we used axial T2-weighted magnetic resonance imaging (MRI) to compare the SAPA between LFS patients and normal controls.

## 2. Methods

### 2.1. Patients

This study was registered at the Eulji University College of Medicine, Republic of Korea. The Institutional Review Board (IRB) reviewed and approved the research protocol. Patients who had visited the Spine Center between March 2014 and April 2016 and were diagnosed with LFS were reviewed retrospectively. Patients > 60 years of age were included if they had clinical manifestations compatible with LFS* (such as neurogenic intermittent claudication, sharp, dull, or radiating pain, numbness or weakness in the lower extremities, sensations of burning, and difficulty standing straight or walking)*, the most stenotic level occurred at L4-L5, and they had MRI imaging performed within 12 months of the diagnosis that was available for review. Patients were excluded if they had a past history of previous spinal injury or lumbar surgery, congenital spine defect, pain disorder, or history of spinal interventions such as kyphoplasty or vertebroplasty.

A total of 137 patients were enrolled after the LFS diagnosis was confirmed by an experienced, board-certified neuroradiologist. In LFS group, there were 66 (48.1%) men and 71 (51.8%) women with a mean age of 71.95 ± 8.05 years (range: 60–88 years) (Table 1). The NRS pain scores were recorded on an 11 cm horizontal line, with 0 indicating no pain and 10 indicating very severe pain. In order to compare the SAPA between patients with and without LFS, a group of control subjects was enrolled. The control patients had undergone lumbar MRI as part of a routine medical examination. Patients in the control group had no LSS-related symptoms. The control group consisted of 167 subjects (85 men and 82 women) with a mean age of 73.85 ± 8.38 years (range: 60–90 years) (Table 1). The SAPA in the control group was similarly examined at the L4-L5 facet joint level.

### 2.2. Imaging Parameters

MRI examinations were performed with 1.5 T (MAGNETOM Aera, MAGNETOM Espree, MAGNETOM Symphony, Sonata, Biograph, Avanto, Siemens Healthcare) and 3 T (MAGNETOM Skyra, MAGNETOM Verio, Siemens Healthcare) scanners. Axial and sagittal T2-weighted images with <3 mm slice thickness were obtained. The following other parameters were used: 0.9 mm intersection gap, 709 ms/12 ms repetition time (TR)/echo time (TE), 30 cm field of view, 448 × 314 matrix, and >3 echo train length (ETL).

### 2.3. Image Analysis

**Figure 1:**
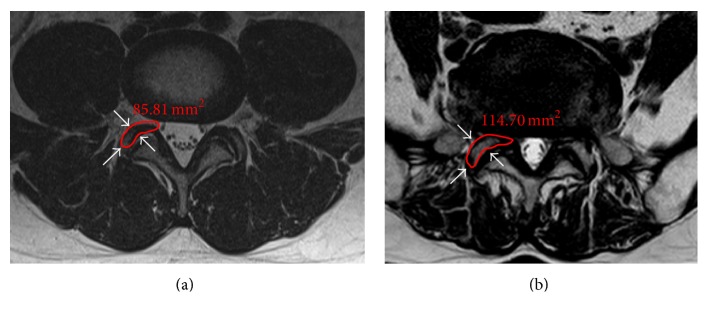
Measurement of the superior articular process area on MRI at the most stenotic level. (a) Control group. (b) Lumbar foraminal stenosis group.

Lumbar foraminal stenosis is defined as when the spinal nerve roots are compressed on the side due to narrowing of the foramen. The axial T2-weighted MR images were acquired at the level of facet joint for individual patient data. The INFINITT image analysis system (INFINITT; INFINITT Healthcare, Seoul, Korea) was used to measure the SAPA at the L4-L5 facet joint level on MRI. The SAPA was measured as the cross-sectional area by outlining the superior articular process at the narrowest foramen (Figure 1).

### 2.4. Statistical Analysis

The data are expressed as means ± standard deviations (SD). Unpaired* t*-tests were used to compare the SAPA between the control and LFS groups. *p* values < 0.05 were considered statistically significant. The validity of the SAPA for diagnosis was estimated using receiver operating characteristic (ROC) curves, optimal cut-off values, area under the curve (AUC), sensitivity, and specificity with 95% confidence intervals (CIs). SPSS version 21 for Windows (IBM SPSS, IBM Corp., Armonk, NY) was used for the statistical analysis.

## 3. Results

**Figure 2:**
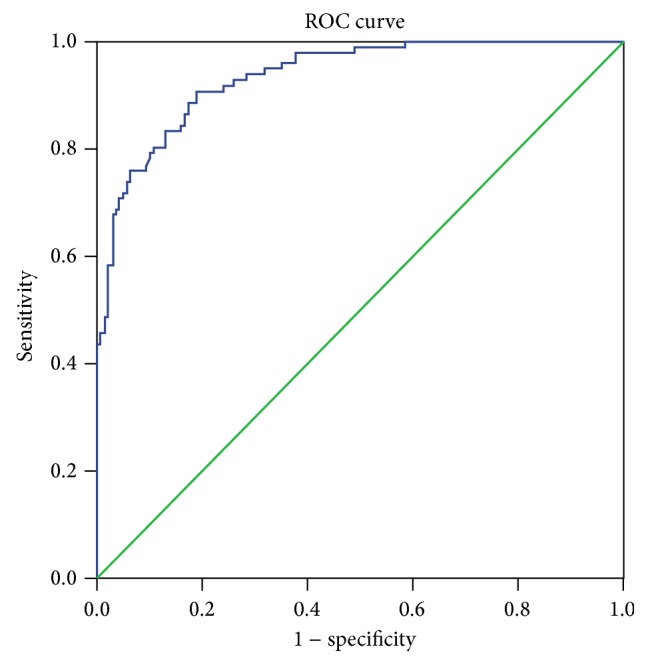
Receiver operating characteristic curve of superior articular process area for prediction of lumbar foraminal spinal stenosis. The best cut-off point of superior articular process area was 112.12 mm^2^, with sensitivity of 84.4%, specificity of 83.9%, and AUC of 0.94.* AUC: area under the curve*.

A total of 167 subjects (85 men and 82 women) were included in the control group. The average age was 73.85 ± 8.38 years. And the average SAPA was 96.31 ± 13.60 mm^2^ in the control group. A total of 137 patients (66 men and 71 women) were included in the LFS group. The average age and VAS score were 71.95 ± 8.05 years and 5.9 ± 1.1. The average SAPA was 128.13 ± 17.23 mm^2^ in the LFS group. There were no significant differences between the groups with regard to age and sex. However, the LFS patients had significantly greater SAPA (*p* < 0.001) than did the control subjects (Table 1). The ROC curve analysis showed that the optimal cut-off point of SAPA was 112.12 mm^2^ with 84.4% sensitivity, 83.9% specificity (Table 2), and AUC of 0.94 (95% CI: 0.91–0.96) (Figure 2). There were no statistically significant correlations between the NRS score and the SAPA.

## 4. Discussion

LSS is the most common spinal disorder in elderly patients that causes low back or buttock pain and intermittent neurogenic claudication [2, 13]. LCSS results from a combination of pathogenic factors, including a decrease in the area of the cauda equina, hypertrophy of ligamentum flavum, loss of intervertebral disk height, and hypertrophy of the facet joints [14]. LFS results from posterolateral osteophytes from the vertebral endplates, which protrude into the foramen along with a herniated disk or a laterally bulging annulus fibrosus. Overgrowth of the facet joint capsule leads to foraminal stenosis [6, 15]. Therefore, facet joint hypertrophy has been considered a major step in the development of LFS. Many previous studies have investigated the associations between the ligamentum flavum, dural sac area, spinal canal area on MRI, and the signs and symptoms of LSS. Park et al. reported that the ligamentum flavum is significantly thinner in patients with intervertebral disc herniation than it is in those with LSS [16]. Altinkaya et al. demonstrated that thickening of the ligamentum flavum was correlated to age, body mass index, and disc degeneration [17]. Ogikubo et al. found that there is a significant relationship between shorter walking distances and a smaller dural sac area [12]. Kim et al. reported that a larger dural sac area is associated with a longer subjective walking distance before the onset of claudication [11]. However, there are no previous reports of an association between LFS and facet joint hypertrophy as a morphologic parameter on MRI. Moreover, there are no objective morphologic parameters that indicate facet joint hypertrophy. Barry and Livesley reported that “facet joint hypertrophy” is a misnomer, because normal facet joints are no smaller than are degenerate facet joints [7]. This group also insisted that there is no clear definition in the literature regarding lumbar facet joint hypertrophy [7, 18]. We believe that, in contrast to facet joint hypertrophy, the SAPA is the precise, objective measurement. Panjabi et al. described the cross-sectional area of the superior articular facet [19]. These cross-sectional areas were analyzed from autopsy specimens. Barry and Livesley measured the cross-sectional area of the superior articular process using computed tomography [7]. In this study, the SAPA was measured from MRI images. To our knowledge, this measurement has not been previously reported. Our results demonstrate associations between the SAPA and LFS. The LFS patients had significantly higher SAPA values than did control subjects. This study only included individuals >60 years of age because previous studies found that facet joints had only minimal cartilage changes before the age of 45 years and that osteoarthritic changes advance with age [8]. Our interpretation of these associations is that hypertrophy may be related to continuous stress, which might increase the SAPA. The process of facet joint hypertrophy begins with mechanical stress during lumbar rotation and flexion. These stressors put force on the facet joints, which leads to a high degree of abrasion [20, 21]. This etiology may alter the morphologic features of the superior articular process. Bajek et al. explained that osteophyte formation in the lumbar spine is an attempt to stabilize an unstable segment; this mechanism ultimately leads to facet hypertrophy [22]. Al-Rawahi et al. have demonstrated that mechanical influences tended to increase with the size of osteophyte. Osteophytes contribute 7% to 9% of the bone mineral density measurement for a motion segment [23]. Disc degeneration may also increase the stressful force on the facet joints [24]. Therefore, osteophytes and hypertrophy of the superior articular process were the main factors of bone tissue [25]. Our results indicate that the visual analog scale score is not significantly associated with the SAPA on MRI. We believe that the pathophysiology of spinal stenosis is multifactorial [26]. Although mechanical compression of the spinal canal is a major factor, subjective discomfort, inflammatory effects [27–29], and depression may also contribute to the disease's pain intensity [30].

This study has several limitations. First, there may be errors associated with measuring the SAPA on MRI. We measured the SAPA in the axial T2 images at the level of the L4-L5 facet joint. However, these axial images may be heterogeneous due to differences in the cutting angle of the MRI resulting from individual anatomic variations and technical problems. A 3.0 mm slice of axial T2-weighted MR image is also thicker than an ideal slice. Second, we did not measure the sagittal cut to calculate the surface area of the superior articular process. Third, degenerative LSS can involve the central canal, the foramina, the extraforaminal zone, or a combination of these locations. However, we focused on LFS. Our analysis would be improved if data including the SAPA of the LCSS and extraforaminal entrapment were compared with our current findings. Fourth, there are several different methods that are known to be effective at discriminating LSS, such as sedimentation sign or morphologic grading [6, 31]. However, this study only used SAPA measurement. Therefore, our results may be limited regarding measurement of the epidural pressure or morphologic changes. Fifth, we excluded the inferior articular process area because this area blurs the boundaries. It is difficult to accurately measure the inferior articular process area in each level due to the overlap. Finally, another limitation of this study is its retrospective nature.

Despite these limitations, this is the first study to document that the SAPA is associated with LFS. These results may help treating physicians recognize an important cause of LFS.

## 5. Conclusions

Our results demonstrate that SAPA is a sensitive measurement parameter for assessing LFS. With regard to LFS, the optimal cut-off value was 112.12 mm^2^, with 84.4% sensitivity, 83.9% specificity, and AUC of 0.94. We believe that this result will help physicians in their evaluations of patients with LFS.

## Figures and Tables

**Table 1:** Comparison of the characteristics of control and LFS groups.

Parameter	Control group (*N* = 167)	LFS group (*N* = 137)
Gender (male/female)	85/82	66/71 (NS)
Age (years)	73.85 ± 8.38	71.95 ± 8.05 (NS)
VAS score		5.9 ± 1.1
SAPA (mm^2^)	96.31 ± 13.60	128.13 ± 17.23 (*p* < 0.001)

Data represent the mean ± standard deviation (SD) or the numbers of patients.

LFS: lumbar foraminal stenosis; VAS: visual analog scale; SAPA: superior articular process area; NS: not statistically significant (*p* > 0.05).

**Table 2:** Sensitivity and specificity of each cut-off point of the SAPA for prediction of lumbar foraminal spinal stenosis.

SAPA (mm^2^)	Sensitivity (%)	Specificity (%)
60.93	100	0
99.82	95.8	62
112.12^*∗*^	84.4	83.9
114.63	65.5	89.8
117.47	58.2	94.9
170.74	0	100

^*∗*^The best cut-off point on the receiver operating characteristic curve. SAPA: superior articular process area.
